# Characterization of Cochlear Implant Artifact and Removal Based on Multi-Channel Wiener Filter in Unilateral Child Patients

**DOI:** 10.3390/bioengineering11080753

**Published:** 2024-07-24

**Authors:** Dario Rossi, Giulia Cartocci, Bianca M. S. Inguscio, Giulia Capitolino, Gianluca Borghini, Gianluca Di Flumeri, Vincenzo Ronca, Andrea Giorgi, Alessia Vozzi, Rossella Capotorto, Fabio Babiloni, Alessandro Scorpecci, Sara Giannantonio, Pasquale Marsella, Carlo Antonio Leone, Rosa Grassia, Francesco Galletti, Francesco Ciodaro, Cosimo Galletti, Pietro Aricò

**Affiliations:** 1 Department of Molecular Medicine, Sapienza University of Rome, Viale Regina Elena 291, 00161 Rome, Italy; giulia.cartocci@uniroma1.it (G.C.); biancams.inguscio@uniroma1.it (B.M.S.I.); gianluca.borghini@uniroma1.it (G.B.); gianluca.diflumeri@uniroma1.it (G.D.F.); 2 BrainSigns srl, Via Tirso 14, 00198 Rome, Italy; vincenzo.ronca@uniroma1.it (V.R.); alessia.vozzi@brainsigns.com (A.V.); fabio.babiloni@uniroma1.it (F.B.); 3 Department of Computer, Control, and Management Engineering “Antonio Ruberti”, Sapienza University of Rome, Piazzale Aldo Moro 5, 00185 Rome, Italy; capitolino.1846005@studenti.uniroma1.it; 4 Department of Anatomical, Histological, Forensic & Orthopedic Sciences, Sapienza University of Rome, Piazzale Aldo Moro 5, 00185 Rome, Italy; andrea.giorgi@uniroma1.it (A.G.); rossella.capotorto@uniroma1.it (R.C.); 5 Department of Physiology and Pharmacology “Vittorio Erspamer”, Sapienza University of Rome, Piazzale Aldo Moro 5, 00185 Rome, Italy; 6 Department of Computer Science, Hangzhou Dianzi University, Hangzhou 310018, China; 7 Audiology and Otosurgery Unit, “Bambino Gesù” Pediatric Hospital and Research Institute, Piazza di Sant’Onofrio 4, 00165 Rome, Italy; alessandro.scorpecci@opbg.net (A.S.); sara.giannantonio@opbg.net (S.G.); pasquale.marsella@opbg.net (P.M.); 8 Department of Otolaringology Head-Neck Surgery, Monaldi Hospital, Via Leonardo Bianchi, 80131 Naples, Italy; carloantonioleone@hotmail.com (C.A.L.); rosa.grassia@ospedalideicolli.it (R.G.); 9 Department of Otorhinolaryngology, University of Messina, Piazza Pugliatti 1, 98122 Messina, Italy; fgalletti@unime.it (F.G.); francesco.ciodaro@unime.it (F.C.); cosimogalletti92@gmail.com (C.G.)

**Keywords:** EEG, cochlear implant, cochlear implant artifact, multi-channel Wiener filter (MWF), artifact reduction

## Abstract

Cochlear implants (CI) allow deaf patients to improve language perception and improving their emotional valence assessment. Electroencephalographic (EEG) measures were employed so far to improve CI programming reliability and to evaluate listening effort in auditory tasks, which are particularly useful in conditions when subjective evaluations are scarcely appliable or reliable. Unfortunately, the presence of CI on the scalp introduces an electrical artifact coupled to EEG signals that masks physiological features recorded by electrodes close to the site of implant. Currently, methods for CI artifact removal have been developed for very specific EEG montages or protocols, while others require many scalp electrodes. In this study, we propose a method based on the Multi-channel Wiener filter (MWF) to overcome those shortcomings. Nine children with unilateral CI and nine age-matched normal hearing children (control) participated in the study. EEG data were acquired on a relatively low number of electrodes (*n* = 16) during resting condition and during an auditory task. The obtained results obtained allowed to characterize CI artifact on the affected electrode and to significantly reduce, if not remove it through MWF filtering. Moreover, the results indicate, by comparing the two sample populations, that the EEG data loss is minimal in CI users after filtering, and that data maintain EEG physiological characteristics.

## 1. Introduction

The absence from birth or loss of hearing constitutes a social barrier, as it limits spoken language development and other fundamental cognitive skills (e.g., memory, reasoning, and visuomotor functions) critical for all social processes involved in social integration [1,2]. A challenging goal for otolaryngologists, speech therapists, audiologists, and neurophysiologists is to combat hearing-related cognitive impairment effectively and relevantly, in order to intervene at the earliest onset of deafness, particularly in typically developing children. In this context, cochlear implants (CI) are neural prostheses that allow deaf patients not only to improve language perception, but they also help to improve its emotional valence assessment, a language feature critical for understanding all the nuances of verbal communication. Extensive literature shows that CI are a valuable tool for supporting and restoring cognitive functions in patients with hearing loss (for a review see [3]) even when hearing aids cannot be used in severe or profound hearing loss cases (above 70 dB hearing threshold).

Typically, after cochlear implantation the device remains inactive for a variable period of time between one and twelve months [4]. Then the device undergoes a programming session to evaluate the patients’ subjective range of stimulation for each electrode on the device in order to establish threshold and comfort levels. This procedure, repeated throughout the rehabilitation, strictly depends on the patient auditory experience and is therefore subjective and prone to under- or overfitting the patients’ needs. In particular, in cases where there is little to no auditory experience prior to the implantation, such as for young children or patients deaf from birth, it is cognitively challenging to evaluate proper hearing levels [2]. Moreover, in children, especially very young ones, where the correct involvement of the supporting family is a crucial aspect [5], their cooperation is not always granted that can even vary from a clinical appointment to the next, challenging a successful rehabilitation. 

Consequently, more objective approaches are needed to support and validate psychoacoustic measures and assist CI fitting. Given the importance of the post-operative period in optimally setting up the cochlear implant, today more and more clinicians are relying on neurophysiology researchers to assess and improve the rehabilitation pathway [6]. EEG is a technique often already present in clinical environments (e.g., surgical rooms) with high temporal resolution, non-invasive and its use does not interfere with the CI, like the intense electromagnetic field generated by MRI, and does not use radioactive tracers as in PET, for which repetitive sessions for CI fitting after surgery are not recommended. Moreover, EEG measures have proven to be helpful in evaluating auditory functions throughout the rehabilitation process, facilitating CI fitting; monitoring speech performance; monitoring brain plasticity post-implantation [7,8,9,10,11]; evaluating listening effort [12,13,14], both in children and adults; and even assisting the initial programming when the patient is still anesthetized [15]. 

Although EEG appears to be the most promising method to support auditory assessments, it comes with a caveat: it is affected by an electromagnetic artifact several orders of magnitude higher than baseline EEG and brain-evoked responses, originated from the CI [2]. A CI device is composed of two main parts: an external sound processor placed on the scalp of the patient and an internal component containing the electrode array that goes inside the cochlea and directly stimulates the auditory nerve. The external audio processor is responsible for acquiring environmental sounds, processing them and ultimately sending the correct pattern of stimulation to the electrode array. The communication with the internal component (through a radiofrequency transmission) and the stimulation of the electrode array implanted in the cochlea are possible candidates for generating CI artifacts in EEG data recording, in particular near the implantation site [16,17,18]. CI artifacts can be time-locked and overlap EEG signal, leading to erroneous detection of brain activity due to its time and phase distortion [19], and therefore hindering its use for clinical purposes (e.g., during surgery or for fitting procedures) and biasing statistical results in auditory studies involving CI users.

Several methodologies have been developed to deal with CI artifact, some of them aim to avoid the presence of the artifact on the EEG segment of interest by using short stimuli that end before target neural activation [19] or by inserting gaps inside the stimulation to reduce the amplitude of the artifact [20]. Other aims to reduce CI artifacts with different preprocessing strategies after the EEG data have been acquired (for a review, see [2]). Simple filtering techniques (low-pass, high-pass, and band-pass) are the first general attempts at removing EEG artifacts; however, they are not effective when the frequency bands of interest are contaminated by the artifact. Thus, alternative techniques have been applied to EEG signals, such as adaptive filtering [21], regression [22], wavelet transform [23], and blind source separation (BSS) techniques, with the most common being independent component analysis (ICA) [24,25,26,27] and ICA and wavelet transform in combination (WICA) [28,29,30].

Regression and adaptive filtering methodologies rely on the availability of a reference signal uncorrelated with EEG data to subtract from the contaminated signal, a condition that is not met in the presence of CI artifact, since the effective characteristics of the artifact could depend on the stimulus, or the processing device implanted. Methodologies that rely on wavelet transform are unable to remove artifacts that overlap the spectral domain of the EEG signal and a threshold criterion is often added [25,31]; moreover, the choice of the mother wavelet is crucial for the effective component to identify, limiting their use to conditions in which the artifact to remove is known or it is possible to have large datasets to apply more complex techniques that involve artificial intelligence [32,33]. ICA relies on the statistical independence of the sources, and even if in EEG the sources are not completely independent, this methodology can be used to effectively identify and separate artifact components from the EEG signal of interest. The drawback of ICA is that it is a data-consuming technique, since large quantities of data should be given to the algorithm to perform optimally, and the maximum number of components that it can separate is constrained by the number of acquiring channels used. Thus, in setups where the number of electrodes is limited, an identified artifactual component still could retain relevant EEG information that will be lost once the component is removed. Considering that a CI artifact can be composed of up to 11 independent components [26], this approach with CI users appears to be limited to those configurations that uses a large number of scalp electrodes. Approaches that combine wavelet transform and ICA probably cannot overcome the limitation posed by the single techniques used alone when dealing with CI artifacts, and to the best of our knowledge, there is still no attempt reported in the literature.

Although the above-discussed methodologies have been effectively used in dealing with CI artifacts [20,24,34,35] and have already been proven efficient also for several other EEG-related artifacts, they pose limitations on their use for the reduction of CI artifacts. On the contrary, Wiener filtering is a parametric technique that can be efficiently applied to biological signals [36]. This filtering technique is based on a statistical approach that does not require an external reference signal and assumes that the artifact and the signal are stationary linear stochastic processes uncorrelated with each other, with known autocorrelation and cross-correlation. The Wiener filter produces a linear time-invariant filter that minimizes the mean squared error between desired (or artifact-free) and estimated (reconstructed) signals, with the artifact estimated from the measurement [31]. The method is semi-supervised since it requires the user to mark, prior to the method application, artifactual segments in order to effectively train the filter to remove only the target artifact. In particular, the Multi-channel Wiener filter (MWF) [36,37,38] used for the removal of ocular artifacts from EEG data takes into account the information from all the recording channels and shares the characteristics of an ideal method for effectively dealing with CI artifact.

Given the above-mentioned reasons, in the present study, we propose a novel use of MWF to reduce CI artifact in EEG data recordings when acquiring data with a relatively low number of channels and during an ecological emotional recognition task, on a cohort of children with CI. To the best of our knowledge, a similar or comparable approach, considering the limitations posed by our particular experimental setup, has not yet been investigated. We base our proposed method on the assumption that the EEG electrodes are less affected by CI artifact once moved further from the implant site enhancing signal-to-noise ratio; thus, we will try to reduce the CI presence on the EEG channel ipsilateral and closest to CI (labeled as the artifactual segment), while using its contralateral electrode as a template for a signal where EEG is less affected by CI. We work on the hypothesis that the EEG content of the two contralateral channels should be similar, not identical, to the extent of normal physiological differences due to hemisphere lateralization, and the CI artifactual component present in the ipsilateral channel that should be excluded from the signal. 

## 2. Materials and Methods

### 2.1. Sample Population

In the present study, a total of 19 children were enrolled: 9 participants were unilateral CI users (UCI; 5 females, 4 males; M_age_ = 9.47 ± 2.33), 7 with the implant on the right side, while 2 had the implant on the left side, all with no hearing aid in the contralateral ear; 10 participants with normal hearing, with age matching those of the UCI group, were used as control group (NH; 6 females, 4 males; M_age_ = 10.95 ± 2.11). In the UCI group, the period of deafness was 5.02 ± 3.67 years on average and onset and etiology varied within the group. One participant for the NH group was removed from the analysis due to poor general EEG signal quality, lowering the number of total participants to 18. All CI users were implanted with Cochlear Limited devices and had no other hearing aid device.

The study was conducted according to the principles outlined in the Declaration of Helsinki of 1975, revised in 2000, and was carefully explained to the participants and to their parents, who signed an informed consent to allow the children participation. The study was approved by the Bambino Gesù Pediatric Hospital Ethic Committee, protocol 705/FS.

### 2.2. Protocol

In order to evaluate the presence of the CI artifact both in a resting condition, namely when no sound other than environmental noise was present, and in the presence of an auditory stimulus to discriminate, the participants were involved in two experimental tasks. In the first task, they were asked to stay quietly in front of a laptop pc for one minute with no sound other than room background environmental noise. In the second task, they were engaged in an emotional recognition task in which a nonverbal vocalization from a previously validated database [39,40], including children [41], was played to the participants and they had to categorize it into three emotional states (positive, negative or neutral) by selecting, through the keyboard, the matching corresponding emotional image presented on the screen. Participants responded to a total of 60 emotional sounds (1.5 s average duration) in a pseudorandomized order and were trained before performing the emotional recognition task to familiarize themselves with the experimental protocol. The performance results from the second task are outside the scope of the present study since we are interested only in the sound-activated artifact produced by the stimuli presented to the Ci users, while we will report the neurophysiological results to verify if the proposed methodology retains EEG data from which it is possible to derive objective measures. For these reasons, we will refer to the first task performed as the rest condition, while we will refer to the emotional recognition task as the sound condition throughout the text.

### 2.3. Data Acquisition and Preprocessing

During both experimental conditions, EEG data were recorded by a portable EEG system (BeMicro, EB neuro, Florence, Italy). A 16-channel cap was used to acquire neurological data (Fpz, Fz, F3, F4, F7, F8, Cz, T3, T4, Pz, P3, P4, P7, P8, O1, and O2) and was placed accordingly to international 10/20 system, with a sampling rate of 256 Hz and impedances kept below 10 kΩ. The ground electrode was positioned on the forehead, while references were placed on both linked earlobes.

In CI users, particular attention was given to electrodes on the ipsilateral side of implant site and, in order to avoid placing electrodes on the CI, some electrodes were excluded from the recording session (Table 1), particularly from temporal and parietal areas. 

Once obtained, EEG data were zero-phase bandpass-filtered with a fifth-order Butterworth digital filter between 2 and 40 Hz in order to obtain information for specific EEG bands of interest: theta, alpha, beta, and lower gamma. Although filtering can be aconsidered a method for CI artifact reduction, since it can be composed by a high-frequency component [42], it is not sufficient to correctly remove possible lower-frequency components still present after filtering. 

**Figure 1:**
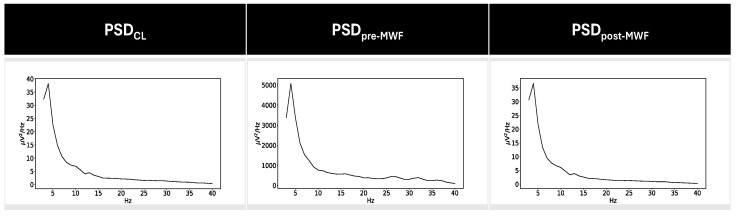
Averaged PSD values for contralateral channel and the channel ipsilateral to CI before and after the application of the proposed method in the UCI group. It is easy to note the higher magnitude of PSD values for PSD_pre-MWF,_ indicating the presence of the CI artifact that covers the underlying EEG data.

From the filtered data, two datasets were obtained, one for the rest condition and one for the sound condition. In the sound condition, only the portion of the recording related to the stimuli presentation during the emotional recognition task was included. Moreover, PSD was obtained from each dataset and for each channel by means of Welch’s method with a 256-points Hamming window with 25% overlap. The PSD of the electrode ipsilateral to the CI that still has the presence of the CI artifact will be referred to as PSD_pre-WMF_, while the PSD relative to its contralateral side will be referred to as PSD_CL_ (Figure 1).

### 2.4. CI Artifact Removal

**Figure 2:**
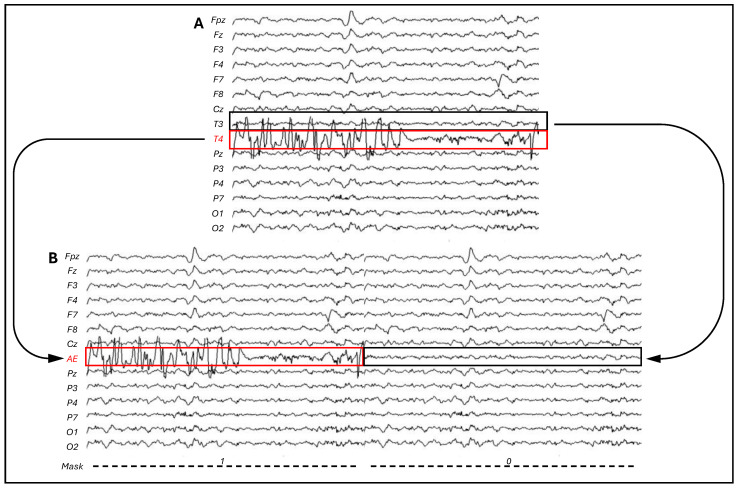
(**A**) *N* channels of original EEG data, with the CI contaminated electrode highlighted in red. (**B**) *M* (*N* − 1) channels EEG data with AE channel (highlighted in red), composed in the first half by the CI contaminated electrode (T4 in the current example) and in the last half by its contralateral electrode (T3). The corresponding mask indicates with 1 where the CI is prominent and 0 where the CI artifact affects the EEG data less.

In order to correctly identify the artifactual component on the EEG data in CI users, we annotated the whole electrode exhibiting the CI artifact, identified by visual inspection (Figure 1), as the artifact segment, while its contralateral electrode was annotated as the non-artifact segment. Then, two EEG datasets were generated and subsequently concatenated: both had *M* = *N* − 1 number of electrodes (with *N* being the total number of electrodes employed on the subject). In one dataset, the electrode with the non-artifact segment was removed, while in the other dataset, the electrode with the artifact segment was removed. All the other electrodes remained the same. The two datasets were then concatenated forming a unique EEG recording with *M* electrodes and double the length of the tasks, in which an artificial electrode (AE) is now present: in the first half, it contains the artifact segment, while in the second half, it contains the non-artifact segment (Figure 2).

The obtained *M*-channel EEG signal y(*t*) ∈ R^M^ at sample time *t* can be modeled as

y(*t*) = n(*t*) + d(*t*), (1)

where n(*t*) ∈ R^M^ represents the neural signal, while d(*t*) ∈ R^M^ represents the non-neural artifact component superimposed to the true neural signal, in our case putatively due to the cochlear implant. We then applied the MWF as a purely special filter as described in [36], exploiting the spatial distribution of the underlying sources (including the CI source) to obtain the estimated full neural component (n) and the CI artifact component (d) related to the first half of AE where the CI artifact was prevalent.

The same procedure has been applied to the EEG data from NH participants to understand if this approach is conservative in respect to EEG signal, thus understanding how much EEG information is lost by applying MWF on CI users. In the NH population, the choice of the artifact segment and its relative contralateral non-artifactual segment has been determined randomly, although mirroring the electrodes identified in the CI user population. Moreover, PSD from n and d has been obtained as described in Section 2.3 and labeled as PSD_post-MWF_ and PSD_artifact_, respectively. 

### 2.5. Similarity Evaluation

From the PSD of the electrodes taken into account and the PSD estimated from the CI artifact component, we obtained their spectral characterization in theta, alpha, beta, and lower gamma bands, the most common EEG bands used for CI users’ assessment [12,43], both for rest and sound condition. 

Moreover, to assess the similarity between the electrode where the CI artifact was present, pre and post MWF filtering, its contralateral electrode and the estimated CI artifact, a series of root mean square error (RMSE) measures was calculated (Table 2) between their respective PSD as follows:

(2) $$RMSE=\sqrt{\frac{\sum_{i=1}^{N}{\left({PSD}_{x}(i)-{PSD}_{Y}(i)\right)}^{2}}{N}}$$

where N is the number of PSD bins.

### 2.6. Neurophysiological Evaluation

In order to understand if the EEG data obtained after the application of MWF retain objective measures to use in the UCI population, we focused on the low gamma band (30–40 Hz), in particular on the right hemisphere, linked to emotion recognition [44,45].

After MWF filtering in CI users, the PSD in the low gamma band (PSD_γ_) was obtained for three seconds after the sound stimulus onset (which includes the full sound and the initial moments of emotion recognition) from the right and left electrodes targeted by the filter (Table 1). In NH group, the target electrodes were T4 and T3, as these represent the most contaminated electrodes in the UCI group. PSD_γ_ obtained from the left and right electrodes was normalized by subtracting the low gamma activity obtained during the resting condition for the corresponding electrodes. Finally, an asymmetry score (AS) was obtained as follows:

$$AS={\left(norm\;{PSD}_{\gamma }\right)}_{right}-{\left(norm\;{PSD}_{\gamma }\right)}_{left}$$

## 3. Results

In this section, we report the results obtained after the application of WMF to CI affected electrodes. In particular, we report the CI artifact characterization and its attenuation after the application of WMF methodology. Moreover, we show how the obtained filtered signal differs from the artifact component extracted and, on the contrary, appears to be more similar to the contralateral electrodes comparing their relationship to the one between contralateral electrodes in NH subjects. Finally, we report the results on the emotional recognition task performed during the sound condition to demonstrate that the EEG after MWF application is still useful for obtaining objective neurophysiological measures. When multiple tests are reported, Bonferroni correction is applied.

### 3.1. CI Artifact Characterization

We compared the artifact component d obtained after the application of MWF to electrodes without CI artifacts (NH group) and to electrodes contaminated by CI artifacts (UCI group), through the use of the Mann–Whitney test for independent samples on their relative PSD data. Each band of interest was taken into account (theta, alpha, beta, and low gamma) both in rest and sound conditions. The results show that the artifact is present in all bands (Table 3) with expected significant higher values for the UCI patient both in rest and sound conditions (all W = 0 and all corrected *p* < 0.001). Moreover, no significant increase in the sound condition compared to the rest condition in UCI patients was highlighted by the Wilcoxon test for paired samples (all corrected *p* > 0.05), indicating the presence of the CI artifact irrespectively to the presence of a target sound.

### 3.2. CI Artifact Reduction

A series of Wilcoxon tests for paired samples between RMSE_pre-CL,_ RMSE_pre-artifact_, RMSE_post-artifact_, RMSE_post-CL,_ and RMSE_CL-artifact_ for UCI is reported in this section, both for the sound condition (Table 4) and rest condition (Table 5), to illustrate how the MWF filtering restores EEG signal on the CI artifact-contaminated electrode. 

**Figure 3:**
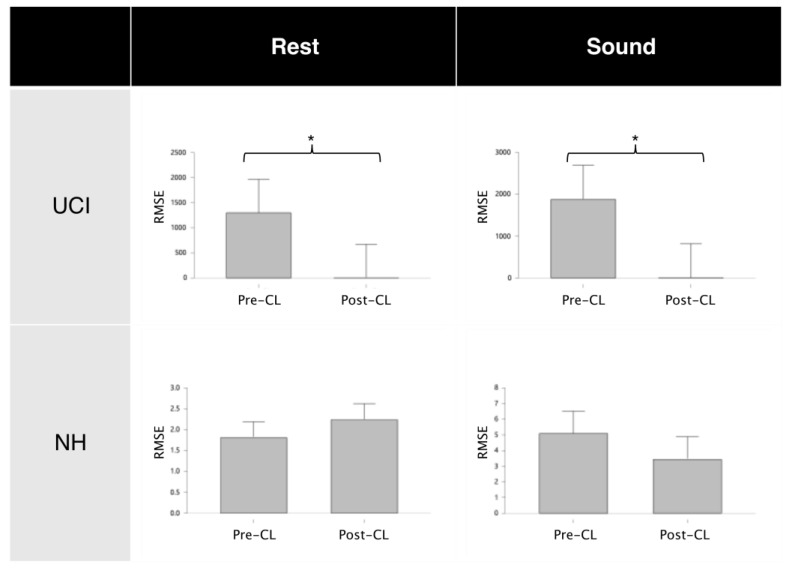
Wilcoxon test results for contralateral electrodes similarity before and after MWF application, showing that MWF application affects only the artifact component (UCI group) and the EEG signal loss is minimal (NH group). Results are shown for both rest and sound conditions. * denotes a significant difference with corrected *p* < 0.001.

The result of the test between RMSE_pre-artifact_ and RMSE_CL-artifact_ in the sound condition indicates that the PSD of CI ipsilateral electrodes is more similar to the PSD from the CI artifact extracted after the application of MWF than the PSD from the contralateral electrodes, as highlighted by the higher value of RMSE between the contralateral electrodes PSD and the artifact PSD (RMSE_pre-artifact_ = 40.12 ± 25.14, RMSE_CL-artifact_ = 1848.31 ± 3459.66; z = −2.67; *p* = 0.03). Moreover, the test between RMSE_pre-artifact_ and RMSE_post-artifact_ reported a significant decrease in similarity between PSD of CI ipsilateral electrode and artifact PSD after the application of WMF (RMSE_pre-artifact_ = 40.12 ± 25.14, RMSE_post-artifact_ = 1849.11 ± 3459.67; z = −2.67; *p* = 0.03). The results from the test between RMSE_pre-CL_ and RMSE_post-CL_ reported an increase in similarity between contralateral electrodes PSD (Figure 3) after MWF filtering, suggesting that the method affects the presence of CI artifact in noisy channels (RMSE_pre-CL_ = 1875.24 ± 3458.53, RMSE_post-CL_ = 2.51 ± 2.16; z = 2.67; *p* = 0.03). Finally, the test between RMSE_post-CL_ and RMSE_post-artifact_ showed a decrease in similarity between the PSD of CI ipsilateral and the artifact PSD and an increase in similarity between ipsi- and contralateral electrodes PSD after the application of MWF, highlighted by the significantly higher values of RMSE_post-artifact_ (RMSE_post-CL_ = 2.51 ± 2.16, RMSE_post-artifact_ = 1849.11 ± 3459.67; z = −2.67; *p* = 0.03). 

Similar results have been obtained for the rest condition. Specifically, the RMSE between the contralateral electrodes PSD and the artifact PSD is significantly higher than the RMSE between the PSD of the CI ipsilateral electrode and the artifact PSD (RMSE_pre-artifact_ = 15.42 ± 16.41, RMSE_CL-artifact_ = 1286.66 ± 2845.83; z = −2.67; *p* = 0.03); the RMSE between the CI ipsilateral electrode PSD and the artifact PSD statistically increases after MWF application (RMSE_pre-artifact_ = 15.42 ± 15.41, RMSE_post-artifact_ = 1287.20 ± 2845.88; z = −2.67; *p* = 0.03), while the RMSE between the contralateral electrodes PSD statistically decreases (RMSE_pre-CL_ = 1299.13 ± 2846.49, RMSE_post-CL_ = 0.94 ± 0.48; z = 2.67; *p* = 0.03). Finally, the RMSE values between contralateral electrodes PSD are significantly lower after the application of the MWF method than RMSE values between the PSD of the CI ipsilateral electrode and the artifact PSD (RMSE_post-CL_ = 0.94 ± 0.48, RMSE_post-artifact_ = 1287.20 ± 2845,88; z = −2.67; *p* = 0.03). 

To check if the changes in similarity between contralateral electrodes were due solely to the application of MWF method, we performed a specular statistical analysis on the NH group. The results highlight that the similarity between electrodes after the application of MWF method remains unaffected (Figure 3) both in sound condition (RMSE_pre-CL_ = 5.08 ± 5.39, RMSE_post-CL_ = 3.43 ± 2.17; z = −0.30; *p* = 0.82) and in rest condition (RMSE_pre-CL_ = 1.81 ± 1.54, RMSE_post-CL_ = 2.24 ± 1.72; z = −0.53; *p* = 0.65), indicating that MWF application not necessarily induces a statistically divergent similarity between electrodes, provided there is no source of artifact present (CI artifact in our case). Moreover, this result also shows that the EEG information lost when applying the MWF method is negligible. 

**Figure 4:**
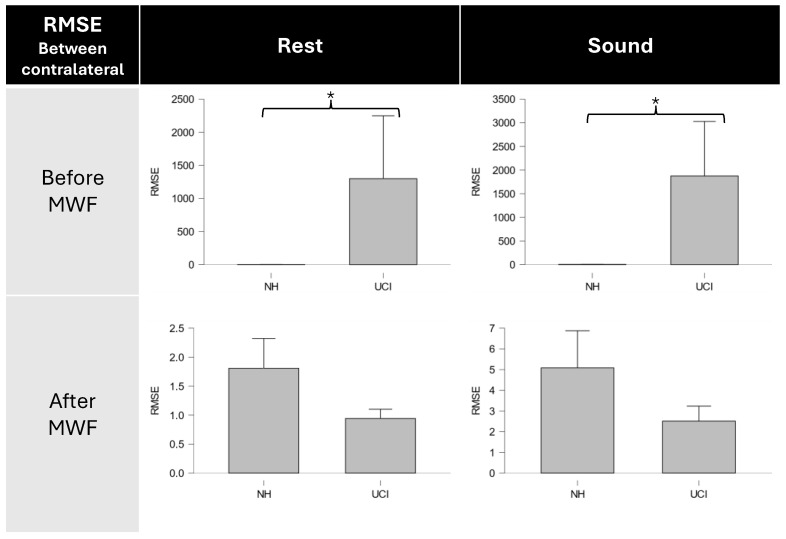
Mann–Whitney test results for contralateral electrodes similarity before and after MWF application in NH and UCI groups, showing comparable relationship between contralateral electrodes after MWF application. Results are shown both for rest and sound conditions. * denotes a significant difference with corrected *p* < 0.01.

Moreover the Mann–Whitney independent sample test (Figure 4) performed between NH and UCI groups in the sound condition, shows that before MWF method, there was much less similarity between contralateral electrodes in the UCI group (NH = 5.08 ± 5.39; UCI = 1875.24 ± 3458.53; W = 0; *p* < 0.001), while after filtering, the similarity between contralateral electrodes in the UCI group is comparable to the one present in the NH group (NH = 5.08 ± 5.39; UCI = 2.51 ± 2.16; W = 56; *p* = 0.76). 

Similar results were obtained in the rest condition, with significantly higher values of RMSE between contralateral electrodes PSD in the UCI group compared to NH participants (NH = 1.81 ± 1.54; UCI = 1299.13 ± 2846.49; W = 0; *p* < 0.001), while no significant difference between groups were obtained after the application of the MWF method (NH = 1.81 ± 1.54; UCI = 0.94 ± 0.48; W = 55; *p* = 0.88). 

### 3.3. Neurophysiological Results

**Figure 5:**
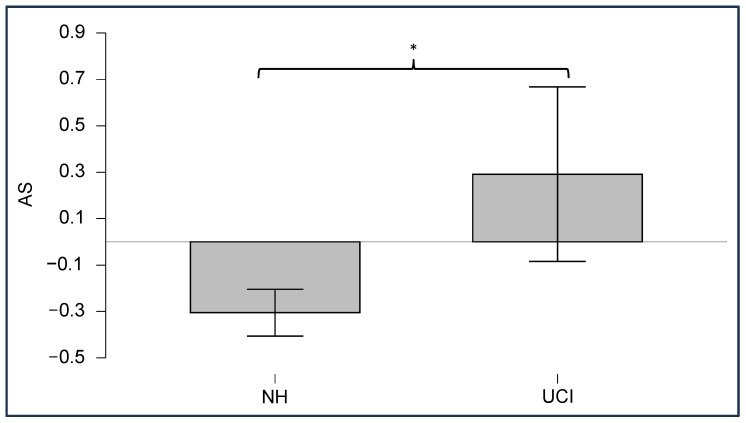
Mann–Whitney test results for the asymmetry score (AS) in NH and UCI groups, showing a significant increase in asymmetry in the UCI population. * denotes a significant difference with *p* < 0.01.

In order to check if the EEG data after the MWF application still retain the characteristics useful to obtain objective measures, we performed a Mann–Whitney independent sample test between NH and UCI groups for AS (Figure 5). The obtained results showed, as expected, a significant higher asymmetry in the low gamma band in the UCI population (NH = −0.31 ± 0.30; UCI = 0.29 ± 1.13; W = 13; *p* = 0.007), indicating higher activity on the right electrodes compared to the left ones during an emotion recognition task. The same analysis was performed on the same set of electrodes for the UCI group before MWF filtering, but no significant results emerged (*p* > 0.05).

## 4. Discussion

The results obtained from CI users and normal-hearing participants indicate that by applying the MWF methodology on EEG contaminated by CI artifacts, it is possible to remove such artifacts and maintain EEG signal information. Additionally, the method allowed one to spectrally characterize the CI artifact in order to understand how it affects the EEG bands relevant for evaluating mental states of CI patients [12,46] during both active listening and resting conditions without the presence of a target sound. Notably, the results showed that the artifact can be observed in both conditions, indicating that the presence of the CI artifact, originating from the devices used in this study, seems to be generated solely by the presence of an active and scanning CI. 

The analysis of EEG data in CI users is inherently constrained by the presence of CI artifacts in the recorded data. Thanks to the MWF application, in the present study we were able to mitigate the limitations posed by the possible use of the state-of-the-art methodologies when dealing with CI artifacts [19,20,24,30,34,35], in particular for EEG data recordings, where a relatively low number of channels are used, and during an ecological task. In fact, in order to approach the reduction of the CI artifact in the present study, using BSS techniques was not advisable due to the risk of removing the EEG signal of interest present in the removed component [24,25,26,27], or the use of methodologies that rely on wavelet transform, since it would be deemed necessary to determine an a priori threshold [25,31], a condition that is not possible to meet when the temporal and spectral characteristics of the artifact we are dealing with are not known. In contrast, given its spatial characteristics, the MWF exploits all the available electrodes in the acquired EEG to train the filter, unlike the classical Wiener filter, and apparently does not require a large number of electrodes as for the BSS methodologies. Moreover, we showed how the MWF application allowed us to obtain an EEG signal as clean as possible, while even maintaining physiological relationships between contralateral electrodes from which it is possible to obtain objective measures (Figure 5). 

**Figure 6:**
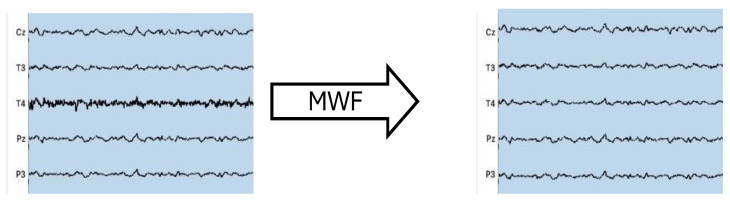
EEG data with T4 channel affected by CI artifact before and after MWF method application, using T3 as noise-free channel. The figure shows how MWF method correctly filters out the CI artifactual component while retaining EEG data.

The analysis of the relationship between contralateral electrodes reported that the electrode ipsilateral to CI has no similarity with its contralateral electrode (Table 4), highlighted by the high values of RMSE, because it is heavily affected by the artifact (Figure 4). After MWF filtering, similarity between contralateral electrodes is restored and the ipsilateral electrode no longer exhibits spectral and temporal characteristics shared with the artifact (Figure 6).

Most importantly, by comparing the data obtained from the UCI group and the data obtained from the NH group, the results showed that the increase in similarity between contralateral electrodes in CI users is not a subproduct of the mere application of the MWF method. In fact, the results support that the filtering effectively removes an artifactual component that is loosely related with EEG data in UC users and that the loss of EEG information, as shown when MWF is applied to artifact-free data in normal hearing participants, is minimal (Figure 3), which is a fundamental prerequisite when dealing with artifact-ridden EEG data. Moreover, the relationship between contralateral electrodes in CI users is greatly affected by the application of MWF. As a matter of fact, the neurophysiological results during the emotion recognition task indicate that the method retains useful EEG information to obtain objective measures to be used on implanted patients (Figure 2) with a higher gamma activity on the right electrode [44,45,47]. Moreover, the proposed methodology restores a more physiological relationship between contralateral electrodes, comparable to the one already present in normal hearing participants during resting condition or during an auditive task (Figure 4).

Summarizing, the method here proposed does not require a previous knowledge of the characteristics of the CI artifact; a test session to train the filter, or a particular methodology to deliver the sound stimuli [19,20], hence appears to be suitable in those experimental paradigms in which an auditory ecological task has to be evaluated by the means of EEG. 

### Limitations and Further Research

Although promising results were obtained in this study, some limitations should be addressed, and further confirmation of the findings is necessary. First and foremost, the proposed method heavily relies on the higher signal-to-noise ratio on the contralateral channel used as artifact-free template; therefore, its application is limited for now to unilateral CI patients, and different approaches should be implemented for bilateral CI patients. We showed how the MWF method can remove, or at least attenuate, the CI artifact on the affected channels, but the CI users that participated in the study were implanted only with devices from a single manufacturer (Cochlear), so further studies must be conducted to possibly extend the application of such a method to CIs from other manufacturers (e.g., Med-El, Advanced Bionics), to understand if the nature of the CI artifact is similar to the one here described. In this regard, we showed that the CI artifact is present both in a condition of quiet, while the CI is only ideally scanning background noise, and while a target sound is present [48,49].

The fact that the CI artifact is present in all EEG bands here taken into account in different auditory conditions could pave the possibility of using the MWF coefficients obtained during a training session to remove the CI artifacts from trial sessions to efficiently monitor in semi-real-time mental states of CI users, such as listening effort [12,13,14,46,50,51,52], crucial for the correct fitting of CI when patients have no auditory experience [53], stress [54,55,56], mental engagement [57,58,59], working memory [57,60,61,62], and emotional processing [47,63].

The application of MWF methodology in the current protocol (on a brief emotional non-verbal vocalization) allows for speculation of its possible use also in other kinds of paradigms in which a more ecological delivery of sound or, even better, during a real conversation, are evaluated. In this regard, we plan to expand the application of such a methodology to other kind of protocol setups.

Moreover, the sample population, in particular the numerosity of the UCI group, could limit a broader generalization of the results; thus, an increase in the number of patients involved in the study (even with different CI devices) could be beneficial to the understanding of the possible application of MWF method on CI artifacts, and even extending the investigation to patients with bilateral CIs.

## 5. Conclusions

The results here reported show that the MWF application to remove or at least attenuate CI artifacts is a valid method that can overcome the shortcomings of current methodologies. In particular, the proposed methodology allows one to maintain to certain degree some EEG physiological information from signal data contaminated by CI artifacts (Figure 5). Its novelty lies in the fact that MWF application does not require modifying the stimulation, allowing for a more ecological protocol (e.g., without the need to insert intervals in the stimuli [20]) and it is possible to use this methodology in recording conditions where a relatively low number of electrodes are used, a condition in which the use of ICA methods is not suitable, due to the possible presence of EEG data in the removed components [24,26,35,36]. Most importantly, the results showed how MWF application restores the physiological relationship between contralateral electrodes when one of them was contaminated by CI artifacts (Figure 4) while still maintaining EEG lateralization (Figure 5), which could be useful for objective measures.

## Figures and Tables

**Table 1:** List of electrodes removed from EEG cap during recording due to CI or incorrect fit on scalp and electrodes in which CI artifact is relevant, along with its contralateral electrode.

UCI Patient	CI Side	Removed Electrode(s)	CI Contaminated Electrode	Contralateral Electrode
P1	Right	P8	T4	T3
P2	Right	T4, p4	P8	P7
P3	Right	F8, P8	T4	T3
P4	Left	Fpz, P7	T3	T4
P5	Left	FZ, P7	T3	T4
P6	Right	P8	T4	T3
P7	Right	P4, P8	T4	T3
P8	Right	P8	T4	T3
P9	Right	P4, P8	T4	T3

**Table 2:** List of RMSE between the PSD of the electrodes taken into account and the PSD obtained from the estimated CI artifact.

PSD_x_	PSD_y_	RMSE Label
PSD_pre-WMF_	PSD_CL_	RMSE_pre-CL_
PSD_pre-WMF_	PSD_artifact_	RMSE_pre-artifact_
PSD_pre-WMF_	PSD_post-MWF_	RMSE_pre-post_
PSD_post-MWF_	PSD_CL_	RMSE_post-CL_
PSD_post-MWF_	PSD_artifact_	RMSE_post-artifact_
PSD_CL_	PSD_artifact_	RMSE_CL-artifact_

**Table 3:** Mean PSD values in theta, alpha, beta, and low gamma in rest and sound conditions for NH and UCI groups. * indicates a significant difference between groups with corrected *p* < 0.001.

Band	Conditions	Groups	Mean (SE)
Theta	Rest *	NH UCI	3.66 (0.75) 2041.97 (1415.53)
	Sound *	NH UCI	9.84 (3.39) 3188.16 (1947.68)
Alpha	Rest *	NH UCI	2.09 (0.31) 765.37 (556.76)
	Sound *	NH UCI	3.33 (1.15) 1351.36 (739.09)
Beta	Rest *	NH UCI	1.16 (0.28) 442.27 (345.20)
	Sound *	NH UCI	1.98 (0.76) 618.42 (415.89
Low Gamma	Rest *	NH UCI	0.41 (0.17) 247.42 (201.24)
	Sound *	NH UCI	0.85 (0.36) 342.82 (219.45)

**Table 4:** Mean RMSE values in the sound condition for the UCI group and relative statistical results. * indicates a significant difference with corrected *p* < 0.05.

	Mean (SE)	z	*p*
RMSE_pre-artifact_ RMSE_CL-artifact_	40.12 (8.38) 1848.31 (1153.22)	−2.67	0.03 *
RMSE_pre-artifact_ RMSE_post-artifact_	40.12 (8.38) 1849.11 (1153.22)	−2.67	0.03 *
RMSE_pre-CL_ RMSE_post-CL_	1875.24 (1152.84) 2.51 (0.72)	2.67	0.03 *
RMSE_post-CL_ RMSE_post-artifact_	2.51 (0.72) 1849.11 (1153.22)	−2.67	0.03 *

**Table 5:** Mean RMSE values in the rest condition for the UCI group and relative statistical results. * indicates a significant difference with corrected *p* < 0.05.

	Mean (SE)	z	*p*
RMSE_pre-artifact_ RMSE_CL-artifact_	15.42 (5.47) 1286.66 (948.61)	−2.67	0.03 *
RMSE_pre-artifact_ RMSE_post-artifact_	15.42 (5.47) 1287.20 (948.63)	−2.67	0.03 *
RMSE_pre-CL_ RMSE_post-CL_	1299.13 (948.83) 0.94 (0.16)	2.67	0.03 *
RMSE_post-CL_ RMSE_post-artifact_	0.94 (0.16) 1287.20 (948.63)	−2.67	0.03 *

## Data Availability

The data presented in this study are available on request from the corresponding author. The data are not publicly available due to privacy reasons.
